# Urethane or Carbamate of Ethyl

**Published:** 1886-04

**Authors:** Henri Huchard, F. R. Campbell


### Translations.

### Urethane or Carbamate of Ethyl.

By Henri Huchard.

Translated from *Bulletin Gen. de Therapeutique*, Feb. 15, 1886, by F. R. Campbell, M. D.

The *urethanes* form a class of ethers derived from carbamic acid, an acid which has not been isolated, but which enters into the composition of several salts and ethers. This acid has certain chemical relations with urea, and it is on this account that the carbamic ethers have been called urethanes.

*Urethane*, or *ethyl-urethane*, is the carbamate of ethyl, or ethylic ether of carbamic acid. Its formula is: $N H^2$, $C O^2$, $C^2$, $H^2$, and is found to consist of rhomboidal crystals, fusing at 55°, and distilling at 180°. It is very soluble in water, alcohol and ether. Its taste is quite agreeable, and resembles somewhat that of nitrate of potash.

This new medicine was introduced into therapeutics by Schmiedeberg, of Strassburg, and has been studied by Jolly, Jacksch of Vienna, and by Riegel and Sticker of Giessen. As a result of numerous experiments upon animals, and clinical observations, these authors were able to recognize a sedative and hypnotic action of this drug. They have shown that, in doses of from one-half a gram to a gram, it will quickly produce a calm sleep, free from dreams. Neither headache nor that dullness observed after the administration of opiates appears. They have also shown that this agent has very feeble toxic properties, and on this account can be administered to children without hesitation. During the past two months I have been studying the action of this medicine, and the results of these experiments I will communicate to you to-day. At my request, my friend, Dr. Eloy, made experiments upon animals, and the following are the results which he obtained:

"In the experiments upon rabbits and guinea pigs, an aqueous solution of urethane was employed. In a rabbit, to whom a dose of one and a quarter grams, three times repeated, were given, the rectal temperature fell from 39.7° to 38.4° in forty-five minutes. At the same time the respirations fell from 90 to 60. The pupils were contracted, sensibility was diminished, particularly in the posterior extremities. Within from twenty-five to thirty-five minutes the animal becomes stupid, totters, and falls on his side, remaining in that position—being in a condition somewhat resembling catalepsy—which lasts for several hours, when the animal awakes apparently unaffected by the drug. In smaller doses the effect is less marked, the temperature is not lowered more than a few tenths of a degree, and respiration is scarcely altered, but the animal appears somewhat stupefied.

"In the guinea pig, after the administration of 35 centigrams of urethane, the temperature fell from 39° to 36° in ten minutes, and motor disturbances appeared in twenty minutes. The disturbances consisted in tottering, falling on the left side, and cataleptiform symptoms. The animal sleeps, but feels painful sensations, in the hind feet slightly, and in the front feet distinctly. After fifty minutes he awakes, and we notice that he has been salivated, sensation is increased, the least movement producing reflex symptoms. There was also hyperæsthesia of the limbs, and the vessels of the ears in both animals were congested during the entire experiment.

"Hence we may assume that even large doses are not poisonous, and that the drug will lower temperature, produce stupor and sleep, and diminish motility and sensation. It will also induce a cataleptiform condition. In the guinea pig, it increases the secretion of saliva, and, on awaking, produces a transitory hyperæsthesia. I will add that one of the rabbits remained in a profound sleep during an entire day, under the influence of 3 grams of urethane, subcutaneously administered. In another experiment, 9 grams were administered to a rabbit without producing death. But we have noticed that hypodermic injections of this drug produce an excoriation in rabbits, which shows that it has an irritant action resembling that of chloral, consequently urethane should not be administered hypodermically."

I have prescribed urethane in fourteen cases suffering from various degrees of insomnia, and affected with various diseases, viz.: angina pectoris, Basedow's disease with aortic insufficiency and pulmonary tuberculosis, parenchymatous interstitial nephritis, mitral affections, cardiac hypertrophy with adherence of pericardium, chronic pulmonary tuberculosis, chronic bronchitis, acute tuberculosis, dyspepsia, and maniacal excitement in a case of paresis.

The following are the results obtained: With the exception of two cases of tuberculosis, with incessant cough and great dyspnœa, all derived benefit from this drug, which produced calm, peaceful sleep without dreams, or digestive disturbance and headache, as sequelæ. Sleep comes on in from ten minutes to one hour after the administration of medicine, and lasts from four to ten hours.

With several patients I made the following experiments: After having given carbamate of ethyl in 3½ gram doses for a few nights, I prescribed a potion having the same taste, but without urethane, and for two days, some of them declared, they had slept perfectly with this draught. This fact may be interpreted in two ways: first, that the hypnotic action of carbamate of ethyl is not very certain; second, that the action of urethane is felt for several days after its administration — to the latter of which I incline.

The dose which I have given to adults is 3 or 4 grams. This dose produces better effects than the 1 or 2 gram doses directed by most German writers. This quantity (3 or 4 grams) should always be taken at one dose, if you wish to obtain a true hypnotic effect; in fact, I always insist upon this as a rule of therapeutics, that all hypnotic medicines should be given in large, undivided doses. I ordinarily prescribe the following potion, to be taken at a single dose in the evening:

| | | Grams. | |
|---|---|---|---|
| ℞ | Urethane, - - - - - | 3 or 4 | — |
| | Syr. Aurant. Cort., - - - | 20 | |
| | Aq. Destillat, - - - | 40 | |

When we wish to give the medicine for several days, I prescribe a solution containing 1 gram to the teaspoonful.

For a child, two months old, affected with bronchitis and insomnia, I ordered, with success, the following potion:

| | | Grams. | |
|---|---|---|---|
| ℞ | Urethane, - - - - - - | 0 | 20 |
| | Syr. Simp., - - - - - - | 20 | |
| | Aq. Aurant. Floris, - - - | 20 | |
| | Aq. Destillat, - - - - | 20 | |

M. Sig.:—Dessertspoonful every two hours.

In a case of Hodgson's disease with acute aortitis, attended with great dyspnœa and obstinate insomnia, I used urethane successfully when hypnone and morphine were absolutely useless. In a child, 8 years of age, affected with acute phthisis, with great dyspnœa and incessant cough, I succeeded in procuring comparatively peaceful sleep with this remedy. It was the same with a woman, 63 years of age, affected with bronchial catarrh, who slept well with doses of from 3½ to 4 grams. In the majority of cases, the restlessness of drunkards, consumptives, and those affected with heart diseases, is successfully overcome by the same remedy. For example: a young patient, 15 years of age, presenting all the symptoms of dilatation of the heart, had for a long time been troubled with a rebellious insomnia. Three grams of urethane succeeded in procuring for him a peaceful sleep, which lasted for eight hours. These facts support those recently published by D. R. Saundry, of London, Eng.

I have never observed any modification of the pressure of the blood in the vessels; but the pulse has *sometimes* been retarded. Schmiedeberg says he has observed a lowering of the arterial tension, while Riegel has noticed quite the opposite. Sticker points out a diuretic action which I have never seen.

Finally, in all my observations, I have never seen urethane produce any unpleasant symptom relating to the stomach, heart, or nervous system. Hence, this remedy may be given with advantage to overcome the restlessness of dyspeptics and those suffering from heart disease, debility, or nervous prostration. For consumptives, urethane is superior to the preparations of opium; and under its influence, dyspnœa and cough are diminished — an experience which has been confirmed by Jacksch and Sticker.

But urethane is greatly inferior to morphine where the insomnia is due to pain or neuralgia. This arises from the fact that urethane is a pure hypnotic, and almost destitute of analgesic properties.

What may we justly conclude from these observations and experiments? In my opinion, a premature conclusion will necessarily be overturned, and we have just learned that we must distrust hasty conclusions, especially when we are dealing with foreign products.*

But urethane is a definite chemical product, and without sharing the enthusiasm of those who would already place this new remedy above chloral, I can say to-day, that in a dose of from three to four grams, urethane has scarcely ever failed to produce a sleep apparently physiological.

This drug will necessarily be of great therapeutic value when we consider the advantages it presents: feeble toxic powers, great solubility in water, a not disagreeable taste, its easy administration to children, the absence of unpleasant sequelæ, and the excellent effects produced in phthisis and heart disease.

Although we do not yet know whether carbamate of ethyl produces anæmia of the brain, it is certain that its physiological action differs from all the opiates which, by inducing a congestion of the nervous centers, produce a pathological sleep. In small doses, opium is an excitant—a fact shown by the celebrated exclamation of Brown: "*Opium, me Hercle non sedat!*" and

*See Hopeine Med. Notes.

recognized by Sydenham, who called it "an excellent cordial." But it is very certain that opium in larger doses produces sleep in an entirely different manner from chloral, paraldehyde, bromide of potassium, or carbamate of ethyl.

The question of hypnotics is again being discussed, thanks to the recent researches relating to paraldehyde, urethane, opium, and acetophenone. This last drug has very unreliable hypnotic powers; but what hypnotic remedy have we to which this reproach does not reply? In the treatment of insomnia we are too forgetful of the causes, which must occupy the first place in selecting our remedies. We forget, too, that we always have hypnotic medications or procedures, but not always reliable hypnotic drugs; that the best means of producing sleep in the feeble, the aged, the convalescent, and the dyspeptic, often consists in nutrition, in restoring the digestive organs, and of giving them at the proper time a good cup of coffee or a small glass of brandy. We have too often endeavored to make physiological sleep depend upon a modification of the cerebral circulation (congestion or anæmia). The brain in sleep is like a muscle in fatigue, which is forced to rest on account of the products of disintegration, which have accumulated in it. Hence all the therapeutic indications established upon the cerebral circulation theory are subject to revision, and it is proper to suggest to investigators a few observations and some useful advice, showing the desiderata of science in regard to hypnotic medication.

